# How did lockdown and social distancing policies change the eating habits of diabetic patients during the COVID-19 pandemic? A systematic review

**DOI:** 10.3389/fpsyg.2022.1002665

**Published:** 2022-09-23

**Authors:** Narges Lashkarbolouk, Mahdi Mazandarani, Farzad Pourghazi, Maysa Eslami, Nami Mohammadian Khonsari, Zahra Nouri Ghonbalani, Hanieh-Sadat Ejtahed, Mostafa Qorbani

**Affiliations:** ^1^Endocrinology and Metabolism Research Center, Endocrinology and Metabolism Clinical Sciences Institute, Tehran University of Medical Sciences, Tehran, Iran; ^2^Non-communicable Diseases Research Center, Alborz University of Medical Sciences, Karaj, Iran; ^3^Obesity and Eating Habits Research Center, Endocrinology and Metabolism Clinical Sciences Institute, Tehran University of Medical Sciences, Tehran, Iran; ^4^Chronic Diseases Research Center, Endocrinology and Metabolism Population Sciences Institute, Tehran University of Medical Sciences, Tehran, Iran

**Keywords:** COVID-19, lockdown, social distancing, eating habits, diabetes

## Abstract

**Background:**

After the declaration of the COVID-19 pandemic, governments established national lockdowns and social distancing as an effective plan to control this disease. As a result of the lockdown policies, diabetic patients` access to food products, medication, and routine follow-ups is disrupted, making it difficult for them to control their disease.

**Methods:**

International databases, including PubMed/Medline, Web of Science, and Scopus, were searched until April 2022. All observational studies included assessing the impact of lockdown and social distancing on eating habits (as primary outcome), and glycemic and anthropometric indices (as secondary outcomes) of diabetic patients during the COVID-19 pandemic. The Newcastle-Ottawa Quality Scale was used to assess the quality rating of the studies.

**Results:**

Overall, 22 studies were included in this systematic review, the results of which varied in different communities. In most studies, consumption of grains, fruits, and vegetables was reported to increase. On the other hand, consumption of snacks and sweets was reported to increase in other surveys. During the COVID-19 lockdown, most diabetic patients preferred to cook meals at home, using less takeout, fast foods, and alcoholic drinks. Although the patients mostly improved their eating habits, the glycemic and anthropometric indices were contradictory in different studies. Studies showed that the eating habits of diabetic patients vary from country to country, even in some cases and studies done in the same country showed different results. For example, all the studies done in Japan showed an increase in the consumption of snacks and sweets, leading to weight gain in the patients. However, conflicting results in eating habits have been observed in studies conducted in India.

**Conclusion:**

The lockdown policies have led to a beneficial change in the eating habits of diabetic patients to consume more fruits and vegetables and reduce the consumption of animal protein products and alcoholic beverages. While some diabetic patients have increased consumption of snacks and sweets, leading to a disturbance in their glycemic and anthropometric indices control. Understanding the consequences of lockdown and social distancing of the diabetic patient during the COVID-19 pandemic can help public health authorities make better recommendations to improve glycemic control.

## Introduction

Since December 2019, millions of people worldwide have been infected with the SARS-CoV2 virus (Ruiz-Roso et al., 2020; Bakaloudi et al., 2021). Due to its nature and airborne transmission, a major step to prevent and control this disease was the impose temporary lockdown plans (Alamri, 2021; Bennett et al., 2021). For this purpose, governments started to close non-essential services such as markets, restaurants, offices, and universities, and people had to stay home. Although the severity of the lockdown policies and their duration varies from country to country, these policies have disrupted the accessibility and availability of food production and supply chains. Also, social distancing and isolation were applied, which led to mandatory changes in lifestyle behaviors especially eating habits. Some countries have relied on voluntary social distancing because they worry that the mandatory lockdown policies may cost too much, even if they reduce health risks. Other countries have imposed strict rules, because either they have seen infection and death rates rise rapidly or because governments have found voluntary levels of social distancing are not enough to keep the pandemic within control (Grabia et al., 2020; Amataiti et al., 2021; Campbell and Wood, 2021).

Chronic diseases like diabetes require constant attention and ongoing treatment to control the disease and prevent complications. Due to the national lockdown policy, routine medical appointments, medication supply, and patient monitoring of the general condition are distorted. Diabetes mellitus (DM) is a chronic metabolic disease, and these patients have high morbidity and mortality from COVID-19 infection owing to metabolic changes and immunosuppression (Palmer et al., 2020; Caruso et al., 2021; Verma et al., 2021).

Diabetic patients need proper eating habits, sleep patterns, mental health, and physical activities to maintain their glycemic control, which can be monitored by HbA1C (hemoglobin A1C) level as an indicator of long-term, fasting blood sugar (FBS), and postprandial blood sugar (PPBS) (Olickal et al., 2020; Khare and Jindal, 2021).

The different economic, cultural, and management differences in lockdown and social distancing in each country have caused the different consequences of changing the lifestyle behavior of diabetic patients. As a result, most studies indicated contradiction in glycemic control during the lockdown and social distancing in diabetic patients (Zupo et al., 2020; Filip et al., 2021). Studies show that eating habits play an important role in blood sugar control (Tiwari et al., 2021). Eating habits can directly affect blood sugar control. For example, in the study of (Kishimoto et al., 2021), positive changes in eating habits help control blood sugar in diabetics.

Overall, there is an information gap about the effect of national lockdown and social distancing on eating habits in diabetic patients. No guidelines have been published regarding the eating patterns of diabetic patients during the lockdown and social isolation. Raising awareness about this condition helps identify new strategies for better health service delivery and management during another pandemic or similar situation. In this systematic review, we aimed to investigate the impacts of the COVID-19 national lockdown and social distancing policies on eating habits (as primary outcome), as well as glycemic and anthropometric indices (as secondary outcomes) in diabetic patients.

## Materials and methods

We followed the preferred reporting items for systematic review and meta-analysis (PRISMA) guidelines to develop the current systematic review (Liberati et al., 2009).

### Search strategy

A systematic literature search was performed on three electronic databases, including PubMed/Medline, Web of Science, and Scopus using standard keywords until April 2022.

The final search string was as following: “eating habits”[Title/Abstract] OR “dietary intake”[Title/Abstract] OR “dietary pattern”[Title/Abstract] OR “food choices”[Title/Abstract] OR “diet quality”[Title/Abstract] OR “eating behaviors”[Title/Abstract] OR “food preference”[Title/Abstract]) AND (“COVID-19”[Title/Abstract] OR “SARS-CoV-2”[Title/Abstract] OR “coronavirus”[Title/Abstract] OR “COVID-19”[Title/Abstract] AND “diabetic patients”[Title/Abstract] OR “diabetics “[Title/Abstract] OR “diabetes”[Title/Abstract] OR “DM Type 1”[Title/Abstract] OR “DM Type 2”[Title/Abstract] OR “Gestational DM “[Title/Abstract].

### Study selection

Two researchers independently screened studies for eligibility based on title, abstract, and full text. Any disagreements between the authors were discussed and resolved by the third author’s opinion.

The inclusion criteria:

1. Studies describe any changes in eating habits as a primary outcome, including consumption of foods and drinks and overall diet quality due to the effect of lockdown and social distancing during the COVID-19 period in diabetic patients.
2. Studies that reported any changes in glycemic indices including (HbA1C, FBS, PPBS) and anthropometric measures such as body mass index (BMI), weight as secondary outcomes due to the effect of lockdown and social distancing in the COVID-19 period in diabetic patients.
3. The population of interest was patients with any type of diabetes.
4. Type of study: Observational studies including cross-sectional, case–control, and cohort.
5. Studies that their full-text were available in English.

The exclusion criteria:

1. Studies that reported eating habits in diabetic patients during the COVID-19 period without comparing it with their previous dietary patterns.
2. Clinical trials, reviews, commentaries, case studies, and letters.

After removing duplicated studies and reviewing the literature independently by two reviewers, disagreements at any screening stage were discussed and resolved by the authors.

### Data collection process

Data regarding the changes in eating habits of diabetic patients during the lockdown and social distancing was retrieved.

The extracted data included:

General characteristics of the included studies (first author name, year of publication, country, study population, and study setting).
Methodological characteristics (study design, sample size, and outcomes assessment method).
Changes in the eating habits and food items in diet (number of meals, amount of meals, change in timing of meals, cooking at home, takeout, eating out, alcohol drinking, snacks, fast foods, sweets, fruits, vegetables, and protein products (meat, egg, fish, and chicken), legumes and grains (rice, cereal, and bread)).
Changes in the glycemic (HbA1C, FBS, and PPBS), and anthropometric indices (BMI, or weight).

### Quality assessment

All included studies were reviewed for quality assessment scores by using the Newcastle-Ottawa Quality Assessment Scale (NOS) for cross-sectional and cohort studies (Wells et al., 2014; Moskalewicz and Oremus, 2020). Two independent investigators assessed the quality of included studies. In case of any disagreement, the third author’s opinion was resolved.

This scale consists of evaluating the methodological quality of the studies in eight items for cohort studies and seven items for cross-sectional studies that contain three categories: Selection of participants (maximum four scores), comparability of subjects (maximum two scores), and assessment of outcome (maximum three scores).

According to quality assessment scales, after calculating scores for cross-sectional studies, we classified 9 and 10 points as “very good,” 7 and 8 points as “good,” 5 and 6 as “satisfactory,” and 0 to 4 as “unsatisfactory.” For cohort studies, “good quality” studies define as if a study achieves 3 or 4 points in the selection part, AND 1 or 2 points in the comparability part, AND 2 or 3 points in the outcome part. “fair quality” studies defined as, if a study achieves two scores in the selection part, AND 1 or 2 scores in the comparability part, AND 2 or 3 points in the outcome part. In addition, if a study gets 0 or 1 in the selection part OR 0 score in the comparability part OR 0 or 1 score in the outcome part, it is considered “poor quality” (Supplementary Tables 1, 2).

### Statistical analysis

Due to heterogeneity between studies in terms of outcomes (eating habits and food items), outcome assessment methods, study design, and setting, the results were synthesized qualitatively and no meta-analysis has been done.

## Results

### Search results and study selection

**Figure 1:**
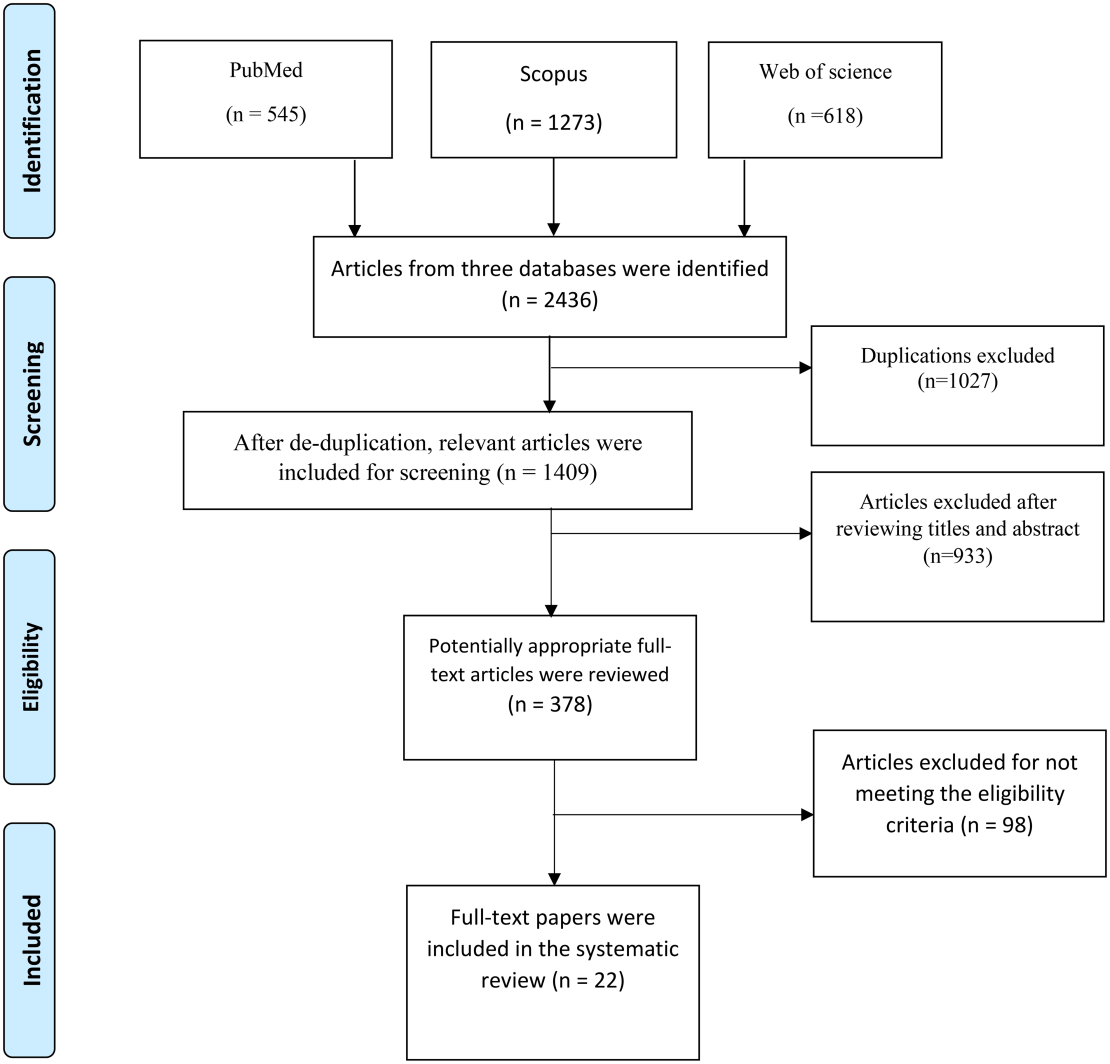
Flow chart of study selection process.

The PRISMA flowchart for study selection is shown in Figure 1. In the initial search of the three databases, 2,436 studies were identified (PubMed/Medline = 545, Scopus = 1,273, Web of Science = 618). After removing 1,027 duplicate documents, 1,409 studies remained. 933 studies were disqualified after reviewing titles and abstracts. 98 studies were withdrawn according to inclusion and exclusion criteria. After that, 378 studies were reviewed in full text, and studies that did not specifically compare eating habits before and during the COVID-19 lockdown and social distancing in diabetic patients were excluded. Finally, 22 studies were selected for this systematic review.

### Study characteristics

A summary of the 22 studies eligible for review is presented in Table 1. Of the 22 studies, thirteen (59%) were cross-sectional (Ghosh et al., 2020; Khader et al., 2020; Munekawa et al., 2020; Olickal et al., 2020; Sankar et al., 2020; Carvalhal et al., 2021; Kishimoto et al., 2021; Sisman et al., 2021; Tanaka et al., 2021; Tiwari et al., 2021; Verma et al., 2021; Maruo et al., 2022; Takahara et al., 2022), and nine (41%) were cohort (Capaldo et al., 2020; Grabia et al., 2020; Khare and Jindal, 2020, 2021; Ruiz-Roso et al., 2020; Amataiti et al., 2021; Caruso et al., 2021; Hansel et al., 2021; Vetrani et al., 2021). All studies were published between 2020 and 2021. The studies were investigated in the different continents such as Europe (Spain = 1, Poland = 1, Italy = 3, France = 1) (Capaldo et al., 2020; Grabia et al., 2020; Ruiz-Roso et al., 2020; Caruso et al., 2021; Hansel et al., 2021; Vetrani et al., 2021), America (Brazil = 1) (Carvalhal et al., 2021), Asia (India = 8, Japan = 5, Turkey = 1)(Liberati et al., 2009; Wells et al., 2014; Capaldo et al., 2020; Grabia et al., 2020; Khare and Jindal, 2020; Moskalewicz and Oremus, 2020; Palmer et al., 2020; Amataiti et al., 2021; Campbell and Wood, 2021; Caruso et al., 2021; Carvalhal et al., 2021; Hansel et al., 2021; Kishimoto et al., 2021; Verma et al., 2021; Vetrani et al., 2021) and one from New Zealand (Amataiti et al., 2021). The total number of diabetic patients for this systematic review was 13,381. Six studies were performed on both types of diabetes (Grabia et al., 2020; Hansel et al., 2021; Kishimoto et al., 2021; Sisman et al., 2021; Maruo et al., 2022; Takahara et al., 2022), and nine studies on type2 diabetes mellitus (T2DM) (Ghosh et al., 2020; Khare and Jindal, 2020, 2021; Munekawa et al., 2020; Olickal et al., 2020; Ruiz-Roso et al., 2020; Sankar et al., 2020; Tiwari et al., 2021; Verma et al., 2021), four studies on type1 diabetes mellitus (T1DM) (Capaldo et al., 2020; Caruso et al., 2021; Carvalhal et al., 2021; Vetrani et al., 2021), and three studies were conducted on any types of diabetes and gestational diabetes mellitus (GDM) (Khader et al., 2020; Amataiti et al., 2021; Tanaka et al., 2021). Due to the restrictions and limitations of the pandemic, most studies used online or phone questionnaires as assessment tools.

**Table 1:** General characteristics of included studies.

First author (Year)	Country	Study setting	Study design	Study population	Sample size	Outcomes	Quality score
Khare and Jindal (2020)	India	Endocrine outpatient department, Questionnaire	Cohort study	Type 2 DM Mean age: 54.68 ± 9.22 yr. Male: 91 Female: 52	*n* = 143	FBS/PPBS number of meals/amount of meals/timings of meals	Fair
Sankar et al. (2020)	Southern India	Outpatient diabetes clinic of MGM Muthoot hospitals, Questionnaire	Cross-sectional study	Type 2 DM Mean age: 58.67 ± 10.8 yr. Male: 42 Female: 68	*n* = 110	HbA1C/weight timing of meals/snacks/vegetables/fruits/fast foods	Satisfactory
Olickal et al. (2020)	Southern India	Diabetes clinic of a tertiary care centre in Puducherry, Questionnaire	Cross-sectional study	Type 2 DM Mean age: 57 ± 12 yr. Male: 274 Female: 76	*n* = 350	FBS/PPBS vegetables/fruits/drinking alcohol	Satisfactory
Ghosh et al. (2020)	India	Telephonic interview	Cross-sectional study	Type 2 DM Mean age: 40-60 yr. Male: 93 Female: 57	*n* = 150	Weight amount of meals/timing of meals/cooking at home/takeout/grains/snacks/fruits/vegetables/sweets/fast foods/protein products	Unsatisfactory
Khader et al. (2020)	India	Online survey	Cross-sectional study	Type 1/2 DM, GDM, and Other types Mean age: 41.6 yr. Male: 963 Female: 547 non-binary: 4	*n* = 1,510	PPBS amount of meals	Satisfactory
Khare and Jindal (2020)	Central India	Endocrinology department of a tertiary care hospital, Questionnaire	Cohort study	Type 2 DM Mean age: 55.68 yr. Male: 181 Female: 126	*n* = 307	HbA1C/FBS/PPBS/weight number of meals/amount of meals/timing of meals	Fair
Ruiz-Roso et al. (2020)	Spain	University hospital La Princesa Madrid, Questionnaire	Cohort study	Type 2 DM Mean age: 64-77 yr. Male: 35 Female: 37	*n* = 72	snacks/vegetables/fruits/ sweets/protein products/legumes	Fair
Caruso et al. (2021)	Italy	Endocrinology division of the university hospital Policlinico Consorziale, Bari, Questionnaire	Cohort study	Type 1 DM Mean age: 42.4 ± 15.9 yr. Male: 25 Female: 23	*n* = 48	PPBS number of meals/grains/vegetables/sweets	Fair
Capaldo et al. (2020)	Italy	Diabetes outpatient clinic of the Federico II university hospital, Naples, Questionnaire	Cohort study	Type 1 DM Mean age: 38.4 ± 12.7 yr. Male: 111 Female: 96	*n* = 207	HbA1C/PPBS amount of meals/timing of meals/snacks	Fair
Grabia et al. (2020)	Poland	Online survey	Cohort study	Type 1/2 DM Mean age: 23 yr. Male: 22 Female: 102	*n* = 124	weight number of meals/cooking at home/takeout/grains/snacks/vegetables /fruits/sweets/protein products/fast foods	Fair
Munekawa et al. (2020)	Japan	Department of endocrinology and metabolism, Kyoto prefectural university of medicine clinic, Questionnaire	Cross-sectional study	Type 2 DM Mean age: 67.4 ± 11.3 yr. Male: 126 Female: 77	*n* = 203	HbA1C/weight amount of meals/takeout/snacks	Satisfactory
Kishimoto et al. (2021)	Japan	Sanno hospital, Questionnaire	Cross-sectional study	Type 1/2 DM Mean age: 62.1 ± 12.3 yr. Male: 116 Female: 52	*n* = 168	HbA1C/BMI amount of meals/timing of meals/cooking at home/takeout/grains/snacks/vegetables/sweets/drinking alcohol	Satisfactory
Takahara et al. (2022)	Japan	Shiraiwa medical clinic, Kashiwara City, Questionnaire	Cross-sectional study	Type 1/2 DM Mean age: 67 ± 13 yr. Male: 863 Female: 539	*n* = 1,402	HbA1C amount of meals/snacks/eating out	Satisfactory
Maruo et al. (2022)	Japan	Department of diabetes, metabolism, and endocrinology of the Osaka police hospital, Questionnaire	Cross-sectional study	Type 1/ 2 DM Mean age: 67.2 ± 11.2 yr. Male: 226 Female: 114	*n* = 340	HbA1C/weight amount of meals/timing of meals/snacks/fruits/grains/drinking alcohol	Satisfactory
Tanaka et al. (2021)	Japan	Kansai electric power hospital, Questionnaire	Cross-sectional study	Type1/2 DM and Other types Mean age: 65 yr. Male: 333 Female: 130	*n* = 463	HbA1C/weight eating out/ takeout/ snacks/ drinking alcohol	Satisfactory
Sisman et al. (2021)	Turkey	Uludag university medical school and medicana hospital endocrinology and metabolism clinic in bursa, Questionnaire	Cross- sectional study	Type1/2 DM Mean age: 42.1 ± 15.5 yr. Male: 169 Female: 135	*n* = 304	weight drinking alcohol/grains/snacks	Satisfactory
Tiwari et al. (2021)	India	Various clinics in Lucknow, uttar Pardesh, Questionnaire	Cross- sectional study	Type2 DM	*n* = 1,406	PPBS	Satisfactory
Verma et al. (2021)	India	Private endocrine clinic in Karnal, Questionnaire	Cross-sectional study	Type 2 DM Mean age: 45 yr. Male: 159 Female: 101	*n* = 260	FBS/PPBS fruits/vegetables/legumes/protein products	Satisfactory
Vetrani et al. (2021)	Italy	Diabetes outpatient clinic of federico II university hospital, Naples, Questionnaire	Cohort study	Type 1 DM Mean age: 38 ± 13 yr. Male: 3 Female: 9	*n* = 12	PPBS protein products/grains/sweets/drinking alcohol	Fair
Amataiti et al. (2021)	New Zealand	Diabetes in pregnancy clinic, Questionnaire	Cohort study	Pregnant women with Type1/2 DM and GDM Mean age: 32.4 ± 5.4 yr. Female: 50	*n* = 50	number of meals/cooking at home/takeout/eating out/grains/snacks/vegetables /fruits/sweets/protein products/fast foods/legumes	Fair
Carvalhal et al. (2021)	Brazil	Online survey	Cross- sectional study	Type 1 DM Mean age: 25–44 yr. Male: 66 Female: 406	*n* = 472	number of meals/amount of meals/cooking at home/takeout/vegetables/fruits/sweets/fast foods	Unsatisfactory
Hansel et al. (2021)	France	Covid IAB web application	Cohort study	Type 1/2 DM Mean age: 52.5 yr. Male: 2587 Female: 2677	*n* = 5,280	BMI snacks/vegetables/ fruits/drinking alcohol	Fair

FBS, fasting blood sugar; PPBS, postprandial blood sugar; HbA1C¸ hemoglobin A1C; BMI, body mass index; DM¸ diabetes mellitus; Type1/2 DM, type 1 or 2 diabetes mellitus; GDM, gestational diabetes mellitus; N.A., not available.

### Changes in eating habits

Table 2 presents the changes in the eating habits of diabetic patients during the COVID-19 lockdown and social distancing. Six of the twenty-two studies reported changes in the number of meals per day, of which four studies reported an increase in the number of meals (Grabia et al., 2020; Khare and Jindal, 2020; Amataiti et al., 2021; Carvalhal et al., 2021) and two studies reported the same number before the lockdown period (Caruso et al., 2021; Khare and Jindal, 2021). For example, in the study of Grabia et al. (2020) and Carvalhal et al. (2021), most participants had five or more meals per day. On the other hand, in the study of Caruso et al., 2021, 72.91% of the participants had the same number of meals as before (Grabia et al., 2020; Caruso et al., 2021; Carvalhal et al., 2021).

**Table 2:** Changes in the eating habits of diabetic patients during COVID-19 lockdown and social distancing.

First author (Year)	Number of meals/day	Amount of meals/day	Changing in the timing of meals	Cooking at home	Takeout	Eating out	Drinking alcohol
Khare and Jindal (2020)	60.14% increase	68.53% increase	60.14% irregular during lockdown	N.A.	N.A.	N.A.	N.A.
Sankar et al. (2020)	N.A.	N.A.	11.8% irregular during lockdown	N.A.	N.A.	N.A.	N.A.
Olickal et al. (2020)	N.A.	N.A.	N.A.	N.A.	N.A.	N.A.	100% decrease in Urban area 62.06% decrease in Rural area
Ghosh et al. (2020)	N.A.	44% decrease 56% no change	42% delayed 13% early 45% no change during lockdown	97% used home -cooked meals	3% used takeout	N.A.	N.A.
Khader et al. (2020)	N.A.	46.88% increase 39.53% no change 13.57% decrease	N.A.	N.A.	N.A.	N.A.	N.A.
Khare and Jindal (2020)	35.5% increase 19.2% decrease 45.3% no change	30.3% increase 21.8% decrease 47.9% no change	51.1% early 23.2% delayed 25.7% no change during lockdown	N.A.	N.A.	N.A.	N.A.
Ruiz-Roso et al. (2020)	N.A.	N.A.	N.A.	N.A.	N.A.	N.A.	N.A.
Caruso et al., 2021	22.91% increase 4.16% decrease 72.91% no change	N.A.	N.A.	N.A.	N.A.	N.A.	N.A.
Capaldo et al. (2020)	N.A.	42% increase 9% decrease 49% no change	26% regular 22% irregular 52% no change during lockdown	N.A.	N.A.	N.A.	N.A.
Grabia et al. (2020)	11.9% increase	N.A.	N.A.	65% started to cook at home during lockdown	10% increase 26% decrease	N.A.	N.A.
Munekawa et al. (2020)	N.A.	24.53% increase	N.A.	N.A.	17.34% increase	N.A.	N.A.
Kishimoto et al. (2021)	N.A.	13.09% increase	2.97% irregular during lockdown	22.61% increase	7.14% increase	N.A.	12.5% increase 20.83% decrease
Takahara et al. (2022)	N.A.	8.3% decrease 7.7% increase	N.A.	N.A.	N.A.	91.2% decrease	N.A.
Maruo et al. (2022)	N.A.	13% increase 11.8% decrease	15.2% regular, 7.5% irregular during lockdown	N.A.	N.A.	N.A.	10.1% increase 24.9% decrease
Tanaka et al. (2021)	N.A.	N.A.	N.A.	N.A.	25.8% increase 13.1% decrease 36.2% no change 24.9% not applicable	2.2% increase 55.8% decrease 19.4% no change 22.6% not applicable	8.7% increase 12.3% decrease 29.5% no change 49.5% not applicable
Sisman et al. (2021)	N.A.	N.A.	N.A.	N.A.	N.A.	N.A.	6.6% stop drinking alcohol
Tiwari et al. (2021)	N.A.	N.A.	N.A.	N.A.	N.A.	N.A.	N.A.
Verma et al. (2021)	N.A.	N.A.	N.A.	N.A.	N.A.	N.A.	N.A.
Vetrani et al. (2021)	N.A.	N.A.	N.A.	N.A.	N.A.	N.A.	40% decrease
Amataiti et al. (2021)	9.37% increase in eating breakfast daily	N.A.	N.A.	42.42% increase	33.33% decrease	55% decrease	N.A.
Carvalhal et al. (2021)	50.4% increase	61.2% increase	N.A.	50.9% increase	46% decrease	N.A.	N.A.
Hansel et al. (2021)	N.A.	N.A.	N.A.	N.A.	N.A.	N.A.	Normal BMI group: 11.7% decrease, 75.8% no change, 12.5% increase high BMI group: 11.6% decrease, 80.8% no change, 7.6% increase

N.A., not available; BMI, body mass index.

A total of 10 studies reported changes in the number of meals, of which six reported an increase (Khader et al., 2020; Khare and Jindal, 2020; Munekawa et al., 2020; Carvalhal et al., 2021; Kishimoto et al., 2021; Maruo et al., 2022), a study reported a decrease (Takahara et al., 2022), and three studies showed no change in the amount of meals for most participants during the lockdown and social distancing (Capaldo et al., 2020; Ghosh et al., 2020; Khare and Jindal, 2021). In a study by Maruo et al., 2022, an increased amount of meals was caused by consuming more carbohydrates, snacks, and fruits. Ghosh et al. (2020) found that the majority of diabetic patients (56%) still maintain the same eating habits as before (Ghosh et al., 2020; Maruo et al., 2022).

Reviewing the studies showed that patients started to eat more home-cooked meals and have fewer takeout, eating out, or fast foods. Four studies reported reduced takeout food consumption (5,6,23,27). There was no change in a study (Tanaka et al., 2021), while an increase was reported in two studies (Munekawa et al., 2020; Kishimoto et al., 2021). In five studies, the patients were asked to eat fast food, of which three reported a decrease (Grabia et al., 2020; Sankar et al., 2020; Amataiti et al., 2021), while two showed an increase (Ghosh et al., 2020; Carvalhal et al., 2021). There was an increase in cooking more at home in five studies (Ghosh et al., 2020; Grabia et al., 2020; Amataiti et al., 2021; Carvalhal et al., 2021; Kishimoto et al., 2021), while three studies announced a decrease in eating out (Amataiti et al., 2021; Tanaka et al., 2021; Takahara et al., 2022). Carvalhal et al. (2021) declared that the reasons for the increased home-cooked meals were financial concerns, changes in food access, taking cooking classes online during the lockdown, and social distancing (Carvalhal et al., 2021).

Changes in the timing of meals were reported in seven studies, of which five studies reported irregular mealtimes (Ghosh et al., 2020; Khare and Jindal, 2020, 2021; Sankar et al., 2020; Kishimoto et al., 2021). In one study, they remained the same as before (Capaldo et al., 2020), while the mealtimes became regular in another (Maruo et al., 2022). In the study of Capaldo et al. (2020), 52% of the participants had the same schedule as before the lockdown (Capaldo et al., 2020).

In seven studies, drinking alcohol during the lockdown and social distancing was investigated. No increase was reported in alcohol consumption in these studies, while a reduction was reported in five studies (Olickal et al., 2020; Kishimoto et al., 2021; Sisman et al., 2021; Vetrani et al., 2021; Maruo et al., 2022), and the trend was the same in the other two studies as before the lockdown (Hansel et al., 2021; Tanaka et al., 2021). Kishimoto et al. (2021) reported that 20.83% of the patients drank fewer alcoholic beverages associated with better glycemic control in them.

### Food items

The changes in the food items of diabetic patients during the COVID-19 lockdown and social distancing are presented in Table 3. Fruits and vegetables consumption increased in six and seven studies, respectively (Ghosh et al., 2020; Grabia et al., 2020; Ruiz-Roso et al., 2020; Sankar et al., 2020; Carvalhal et al., 2021; Maruo et al., 2022) (Ghosh et al., 2020; Grabia et al., 2020; Olickal et al., 2020; Ruiz-Roso et al., 2020; Sankar et al., 2020; Amataiti et al., 2021; Carvalhal et al., 2021). However, two studies mentioned a decrease in fruit consumption (Olickal et al., 2020; Amataiti et al., 2021), and one study reported a reduction in vegetable consumption (Kishimoto et al., 2021). Two (Hansel et al., 2021; Verma et al., 2021) and three (Caruso et al., 2021; Hansel et al., 2021; Verma et al., 2021) studies consumed the same amount of fruits and vegetables. Verma et al. (2021) explained that the increased consumption of fruits and vegetables was because of their positive role in improving the immune system.

**Table 3:** Changes in food items in diet of diabetic patients during COVID-19 lockdown and social distancing.

First author (Year)	Fruits	Vegetables	Snacks	Grains	Fast foods	Sweets	Legumes	Protein products
Khare and Jindal (2020)	N.A.	N.A.	N.A.	N.A.	N.A.	N.A.	N.A.	N.A.
Sankar et al. (2020)	42.7% increase 21.8% decrease	80.9% increase 10% decrease	24.5% increase 63.6% decrease	N.A.	24.5% increase 63.6% decrease	N.A.	N.A.	N.A.
Olickal et al. (2020)	5.7% decrease in Urban area 12.98% decrease in Rural area (at least 1 day/week)	9.6% increase in Urban area 11.4% increase in Rural area (every day/week)	N.A.	N.A.	N.A.	N.A.	N.A.	N.A.
Ghosh et al. (2020)	27% increase 17% decrease 26% no change	9% increase (3 < servings)	70% increase 30% no snacks	21% increase	5% increase	7% increase 88% no sugar 5% no change	N.A.	3% increase 8% decrease 88% not applicable
Khader et al. (2020)	N.A.	N.A.	N.A.	N.A.	N.A.	N.A.	N.A.	N.A.
Khare and Jindal (2020)	N.A.	N.A.	N.A.	N.A.	N.A.	N.A.	N.A.	N.A.
Ruiz-Roso et al. (2020)	1.35% increase in men 10.38% increase in females	47.60% increase (>2 times/w)	96.55% increase	N.A.	N.A.	28.01% increase	5% increase in men, 5.88% increase in females	1.11% decrease in men, 1.54% decrease in females
Caruso et al. (2021)	N.A.	25% increase 18.7% decrease 56.2% no change	N.A.	14.6% increase 14.6% decrease 70.8% no change	N.A.	35.4% increase 16.6% decrease 47.9% no change	N.A.	N.A.
Capaldo et al. (2020)	N.A.	N.A.	35% increase 9% decrease 66% no change	N.A.	N.A.	N.A.	N.A.	N.A.
Grabia et al. (2020)	44% increase 15% decrease	40% increase 11% decrease	19% increase, 29% decrease in salty snacks, 21% increase, 22% decrease in sweet snacks	37% increase, 13% decrease in grains, 31% increase, 6% decrease in homemade bread, 24% increase, 18% decrease fresh bread	14% increase 32% decrease	19% increase 11% decrease	N.A.	22% increase and 14% decrease in fresh fish, 14% increase and 16% decrease in frozen fish, 15% increase and 22% decrease in red meat, 27% increase and 15% decrease in white meat
Munekawa et al. (2020)	N.A.	N.A.	22.28% increase	N.A.	N.A.	N.A.	N.A.	N.A.
Kishimoto et al. (2021)	N.A.	2.97% decrease	25% increase	4.76% increase	N.A.	25% increase	N.A.	N.A.
Takahara et al. (2022)	N.A.	N.A.	15.8% increase 10.1% decrease	N.A.	N.A.	N.A.	N.A.	N.A.
Maruo et al. (2022)	21.8% increase 12.9% decrease	N.A.	15.3% increase 9.1% decrease	22.2% Increase 6% decrease	N.A.	N.A.	N.A.	N.A.
Tanaka et al. (2021)	N.A.	N.A.	21% increase 12% decrease 41.8% no change 25.2% not applicable	N.A.	N.A.	N.A.	N.A.	N.A.
Sisman et al. (2021)	N.A.	N.A.	52.6% increase 47.4% decrease	41.4% increase 58.6% decrease	N.A.	N.A.	N.A.	N.A.
Tiwari et al. (2021)	N.A.	N.A.	N.A.	N.A.	N.A.	N.A.	N.A.	N.A.
Verma et al. (2021)	35% increase 8.8% decrease 55.8% no change	25% increase 4.2% decrease 70.8% no change	N.A.	N.A.	N.A.	N.A.	5.4% increase 3.8% decrease 90.8% no change	0% increase 90% decrease 10% no change in meat and fish, 0.8% increase 50.9% decrease, 45.5% no change in egg
Vetrani et al. (2021)	N.A.	N.A.	N.A.	16.29% increase (in gr)	N.A.	81.25% increase (in gr)	N.A.	21.58% decrease (in gr)
Amataiti et al. (2021)	20% decrease (at least≥4set/day)	10.52% increase (at least≥4set/day)	27.27% increase (at least≥4d/w)	23.07% increase (at least≥4d/w)	100% decrease (at least≥4d/w)	9.09% increase in confectionary, 0% in cakes and biscuits, 42.85% decrease in sugar sweetened beverages (at least≥4d/w)	50% decrease (at least≥4d/w)	37.5% decrease in red meat, 14.28% increase in processed meat, 50% increase in fish (fresh/frozen), 0% in fish battered for at least≥4d/w
Carvalhal et al. (2021)	42.6% appropriate consumption	53.4% appropriate consumption	N.A.	N.A.	31.4% increase	47.9% increase	N.A.	N.A.
Hansel et al. (2021)	normal BMI: 15.4% decrease, 70.4% no change, 14.2% increase high BMI: 17.3% decrease, 67.4% no change, 15.2% increase	normal BMI: 11.8% decrease, 73.7% no change, 14.5% increase high BMI: 14.3% decrease, 69.7% no change, 15.9% increase	normal BMI: 14.3% decrease, 66.5% no change, 19.2% increase high BMI: 19.6% decrease, 59.4% no change, 21.1% increase	N.A.	N.A.	N.A.	N.A.	N.A.

N.A., not available; BMI, body mass index.

Regarding the use of snacks and sweets, eight and five studies stated the consumption of more snacks and sweets by diabetics. According to Maruo et al., 2022, the patients mostly ate more snacks associated with their mental health deterioration (Maruo et al., 2022). Caruso et al., 2021 revealed the increased sweets intake owing to staying more at home (Caruso et al., 2021). On the other hand, two studies reported a decrease in using snacks (Grabia et al., 2020; Sankar et al., 2020) and sweets (Ghosh et al., 2020; Amataiti et al., 2021). Four studies reported no changes in the consumption of snacks and sweets (Capaldo et al., 2020; Hansel et al., 2021; Tanaka et al., 2021) (Caruso et al., 2021).

Diabetic patients also used fewer protein products such as red and white meat, fish, or eggs, as mentioned in four studies (Ghosh et al., 2020; Ruiz-Roso et al., 2020; Verma et al., 2021; Vetrani et al., 2021), while two studies mentioned using more protein sources (Grabia et al., 2020; Amataiti et al., 2021). Verma et al. (2021) reported decreased protein product intake in diabetic patients.

Six studies revealed eating more grains in daily meals (Ghosh et al., 2020; Grabia et al., 2020; Amataiti et al., 2021; Kishimoto et al., 2021; Vetrani et al., 2021; Maruo et al., 2022), one showed a decrease (Sisman et al., 2021), and one recorded no changes in grain usage (Caruso et al., 2021). Sisman et al. (2021) concluded the increased carbohydrate (grain) intake owing to emotional stress in diabetics (Sisman et al., 2021).

There were inconsistent data on using legumes. A study revealed an increase (Ruiz-Roso et al., 2020), another study represented a decrease (Amataiti et al., 2021), and no change was reported in one study (Verma et al., 2021) in this regard. Ruiz-Roso et al. (2020) declared that legumes are considered a superior food group, which must be used by patients in confinement (Ruiz-Roso et al., 2020).

### Glycemic and anthropometric indices

Results of the COVID-19 lockdown and social distancing impacts on glycemic and anthropometric indices in diabetic patients are presented in Table 4. HbA1C level was reported in eight studies, while an increase was reported in two studies (Munekawa et al., 2020; Khare and Jindal, 2021) and a decrease in HbA1C level was represented by four studies (Capaldo et al., 2020; Sankar et al., 2020; Tanaka et al., 2021; Maruo et al., 2022). However, two studies reported no change in HbA1C in most patients during the lockdown compared to the pre-lockdown period (Kishimoto et al., 2021; Takahara et al., 2022). In the studies of Khare and Jindal (2020) and Munekawa et al. (2020), the mean of HbA1C increased by 0.51 and 0.1% in patients. However, Sankar et al. (2020) and Maruo et al., 2022 reported that HbA1C decreased by 0.97 and 2.11% in participants during the lockdown and social distancing (Munekawa et al., 2020; Sankar et al., 2020; Khare and Jindal, 2021; Maruo et al., 2022).

**Table 4:** Glycemic and anthropometric indices.

First author (Year)	FBS	PPBS	HbA1C	Anthropometric indices
Khare and Jindal (2020)	3.88% increase (mg/dl)	34.1% increase (mg/dl)	N.A.	N.A.
Sankar et al. (2020)	N.A.	N.A.	0.97% decrease (%)	0.41% increase in weight (kg)
Olickal et al. (2020)	22.7% (of participants) ideal FBS level during lockdown 14.7% (of participants) satisfactory FBS level during lockdown 62.4% (of participants) unsatisfactory FBS level during lockdown	8.6% (of participants) ideal PPBS level during lockdown 17.3% (of participants) satisfactory PPBS level during lockdown 74.1% (of participants) unsatisfactory PPBS level during lockdown	N.A.	N.A.
Ghosh et al. (2020)	N.A.	N.A.	N.A.	33% (of participants) had decrease in weight 19% (of participants) had increase in weight 48% (of participants)had no change in weight
Khader et al. (2020)	N.A.	78.42% (of participant) increase	N.A.	N.A.
Khare and Jindal (2020)	5.52% increase (mg/dl)	15.59% increase (mg/dl)	0.51% increase (%)	N.A.
Ruiz-Roso et al. (2020)	N.A.	N.A.	N.A.	N.A.
Caruso et al. (2021)	N.A.	1.45% increase (mg/dl)	N.A.	N.A.
Capaldo et al. (2020)	N.A.	1.04% decrease (mg/dl)	0.1% decrease (%)	N.A.
Grabia et al. (2020)	N.A.	N.A.	N.A.	In type1 DM group, 44 and 30% (of participants) had increase and decrease in weight (kg) in type2 DM group, 30 and 29% (of participants) had increase and decrease in weight (kg)
Munekawa et al. (2020)	N.A.	N.A.	0.1% increase (%)	0.3% increase weight (kg)
Kishimoto et al. (2021)	N.A.	N.A.	33.92% (of participants) had increase >0.2% 30.35% (of participants) had decrease>0.2% 35.71% (of participants) had no changes	15.5% (of participants) had increase >2 kg in weight 7.1% (of participants) had decrease>2 kg in weight
Takahara et al. (2022)	N.A.	N.A.	34.9% (of participants) had increase (≥0.3%) 13.4% (of participants) had decrease (≤0.3%) 51.8% (of participants) no change	N.A.
Maruo et al. (2022)	N.A.	N.A.	2.11% decrease (%)	0.29% increase in weight (kg)
Tanaka et al. (2021)	N.A.	N.A.	0.2% decrease (%)	0.14% decrease in weight (kg)
Sisman et al. (2021)	N.A.	N.A.	N.A.	14.8% (of participants) had decrease in weight (kg) 47% (of participants) no changed in weight (kg) 38.2% (of participants) had increase in weight (kg)
Tiwari et al. (2021)	N.A.	60.61% (of participants) had control blood glucose 39.39% (of participants) had uncontrolled blood glucose	N.A.	N.A.
Verma et al. (2021)	9.1% decrease (mg/dl)	5.43% decrease (mg/dl)	N.A.	N.A.
Vetrani et al. (2021)	N.A.	1% (of participants) increase in control blood glucose	N.A.	N.A.
Amataiti et al. (2021)	N.A.	N.A.	N.A.	N.A.
Carvalhal et al. (2021)	N.A.	N.A.	N.A.	N.A.
Hansel et al. (2021)	N.A.	N.A.	N.A.	normal BMI group: 12.8% (of participants) decrease, 63.8% (of participant) no change, 22.9% (of participants) increase in weight high BMI group: 18.9% (of participant) decrease, 51.4% (of participants) no change, 28.6% (of participant) increase in weight

FBS, fasting blood sugar; PPBS, postprandial blood sugar; HbA1C, hemoglobin A1C; BMI, body mass index; DM, diabetes mellitus; Type1/2 DM, type 1 or 2 diabetes mellitus; N.A., not available.

An increase in FBS levels was reported in three studies (Khare and Jindal, 2020, 2021; Olickal et al., 2020), while a study showed a decrease (Verma et al., 2021). The studies of Khare and Jindal (2020) declared that the FBS level increased by 5.52% (mg/dl). In contrast, Verma et al. (2021) reported the decreased FBS levels of the participants by 9.1% (mg/dl) (Khare and Jindal, 2021; Verma et al., 2021).

Six studies revealed increased PPBS (Khader et al., 2020; Khare and Jindal, 2020, 2021; Olickal et al., 2020; Caruso et al., 2021; Vetrani et al., 2021) while two studies reported the decreased one (Capaldo et al., 2020; Verma et al., 2021). Also, one study mentioned that most patients had control of PPBS (Tiwari et al., 2021). In the studies of Khare and Jindal (2020), PPBS levels increased by 34.1% (mg/dl). However, Capaldo et al. (2020)showed a reduction of 1.04% (mg/dl) in PPBS levels in participants (Capaldo et al., 2020; Khare and Jindal, 2020).

Five studies reported an increase in BMI and weight (Grabia et al., 2020; Munekawa et al., 2020; Sankar et al., 2020; Kishimoto et al., 2021; Maruo et al., 2022), while one showed a decrease (Tanaka et al., 2021). In three studies, most patients reported no change in BMI and weight during lockdown compared to the pre-lockdown period (Ghosh et al., 2020; Hansel et al., 2021; Sisman et al., 2021). Maruo et al., 2022 reported that there was a 0.29% increase in weight (kg). However, according to Tanaka et al. (2021), the patients had a reduction of 0.14% in weight (kg) (Tanaka et al., 2021; Maruo et al., 2022).

## Discussion

In this systematic work, we reviewed the studies regarding the impact of lockdown policies and social distancing on the eating habits of diabetic patients during the COVID-19 pandemic. Most studies reported the changes in eating habits of diabetic patients due to national lockdown and social distancing and their tendency to consume healthy meals.

The majority of studies declared healthier diabetic patients’ eating habits during the lockdown and social distancing of COVID-19. Especially, they tended to cook more at home, do less takeout, and eat out as well as use more fresh fruits, vegetables, and grains. Moreover, most of the studies showed a decrease in the consumption of protein products, fast food, and alcoholic beverages. The negative aspects of changing eating habits included increasing the number and amount of daily meals and consuming snacks and sweets.

According to the studies, the increased desire to cook meals at home can be attributed to more free time to cook at home, reduced emotional stress, and fear of exposure to the coronavirus. As a result, the consumption of fast food, takeout, and eating out decreased (Ghosh et al., 2020; Grabia et al., 2020; Sankar et al., 2020; Amataiti et al., 2021; Carvalhal et al., 2021; Tanaka et al., 2021; Takahara et al., 2022). Additionally, trends on social media and online cooking classes seem to support this result (Ghosh et al., 2020; Grabia et al., 2020; Amataiti et al., 2021; Carvalhal et al., 2021; Kishimoto et al., 2021). In the study of Kent et al. (2022), these changes in eating habits were also reported in the normal population during the COVID-19 lockdown and social distancing (Kent et al., 2022). Even though most patients tended to consume more fresh fruits, vegetables, and grains, this inclination faced difficulties such as limited access and availability of fresh food and disruptions in food distribution (Ghosh et al., 2020; Grabia et al., 2020; Olickal et al., 2020; Ruiz-Roso et al., 2020; Sankar et al., 2020; Amataiti et al., 2021; Carvalhal et al., 2021; Kishimoto et al., 2021; Vetrani et al., 2021; Maruo et al., 2022). These may explain why some studies reported a decrease in consumption of fresh fruits, vegetables, and grains (Olickal et al., 2020; Amataiti et al., 2021; Hansel et al., 2021; Kishimoto et al., 2021; Picchioni et al., 2021; Sisman et al., 2021; Verma et al., 2021).

Patients are consuming fewer protein products, possibly for various reasons, including the limited availability of protein products, the higher cost compared to the vegan diets, and the associated economic crisis of the COVID-19 lockdown and social distancing (Ghosh et al., 2020; Zupo et al., 2020; Verma et al., 2021; Vetrani et al., 2021). Regarding legume consumption, there were different outcomes. Unfortunately, few studies have been performed in this regard, and they presented different conclusions. One study reported an increase in the use of legumes, which seems to be due to ease of access and long shelf life compared to other foods (Ruiz-Roso et al., 2020).

Because of confinement, situations like emotional stress, and more time to cook at home, diabetic patients are encouraged to increase the number and amount of meals per day. Besides, working from home allows them to easily eat and drink as they please during working hours (Grabia et al., 2020; Khader et al., 2020; Khare and Jindal, 2020, 2021; Munekawa et al., 2020; Amataiti et al., 2021; Caruso et al., 2021; Carvalhal et al., 2021;Kishimoto et al., 2021; Maruo et al., 2022).

Eating times were also disrupted in these patients. In most studies, patients admitted that their eating times became irregular during the national lockdown and social distancing. Some of them started taking their food earlier or later (Ghosh et al., 2020; Khare and Jindal, 2020, 2021; Sankar et al., 2020; Kishimoto et al., 2021). These irregular mealtimes can easily be linked to an increase in the patient’s body weight or BMI. According to most studies, one of the most significant changes during the lockdown and social distancing was decreased alcohol consumption. Patients tried to reduce drinking alcohol because of affecting the health system. Some studies mentioned that using fewer alcoholic beverages improved glycemic indices (Olickal et al., 2020; Hansel et al., 2021; Kishimoto et al., 2021; Sisman et al., 2021; Tanaka et al., 2021; Vetrani et al., 2021; Maruo et al., 2022).

In contrast to adjusting to healthier eating habits, most study participants started eating more snacks and sweets during the lockdown and social distancing possibly owing to spending more time at home, missing out on social activities, and feeling tired and sometimes bored, even when not hungry (Ghosh et al., 2020; Grabia et al., 2020; Munekawa et al., 2020; Ruiz-Roso et al., 2020; Amataiti et al., 2021; Carvalhal et al., 2021; Hansel et al., 2021; Kishimoto et al., 2021; Sisman et al., 2021; Maruo et al., 2022; Takahara et al., 2022).

Overall, there are two main controversies in the studies’ results. Although the patients consume proper eating habits, they encounter the issue of increased body weight and BMI and the contrast between HbA1C and FBS, PPBS levels as glycemic indices in most studies. Diabetic patients have attempted to manage and control their BMI and body weight. However, most studies have reported an increase in these indices (Grabia et al., 2020; Munekawa et al., 2020; Sankar et al., 2020; Kishimoto et al., 2021; Maruo et al., 2022). As a result, these changing eating habits lead to poor weight control and altered BMI. Most of the participants in the studies took advantage of this lockdown to improve their diet. As a consequence of enhancing their eating habits, it was assumed that these changes could also lead to controlling their body weight and BMI. However, the observations were different in most studies. We must consider some issues in the interpretation of this unexpected outcome. First, many factors, such as physical activity, sleeping patterns, and emotional stress, can influence body weight. According to the results of the studies, patients’ daily physical activity and exercise are affected by the home lockdown and its policies. In other words, they have to stay at home because gyms and sports clubs are closed, thus reducing their physical activity. Second, despite the changes in their eating habits, they still use more snacks and sweets during the lockdown and social distancing as a hobby, which increases their BMI or body weight. Third, it appears that diabetics have been stressed during confinement due to the difficulty in accessing medication and routine monitoring thus affecting their blood sugar control and body weight (Grabia et al., 2020; Munekawa et al., 2020; Sankar et al., 2020; Cena et al., 2021; Kishimoto et al., 2021; López-Bueno et al., 2021; Matsuo et al., 2021; Maruo et al., 2022). In most studies, long-term glycemic indices such as HbA1C in patients with diabetes were improved and had a significant positive effect as a result of healthier lifestyle choices, including eating habits (Capaldo et al., 2020; Sankar et al., 2020; Tanaka et al., 2021; Maruo et al., 2022). However, there was a matter of increasing FBS and PPBS in some studies (Khader et al., 2020; Khare and Jindal, 2020, 2021; Olickal et al., 2020; Caruso et al., 2021; Vetrani et al., 2021). Diabetics use the glycemic indices as an indicator of their blood sugar control. These glycemic indices include HbA1C as a long-term index and FBS and PPBS as short and random indices. In the management of diabetes, blood sugar testing is often essential. Therefore, FBS and PPBS levels in these studies were self-monitored at home, while HbA1C levels were monitored in the laboratory under physician supervision. Due to the wide range of instruments and the use of different sampling methods, the possibility of error in the report is high. On the other hand, HbA1C levels indicate average blood sugar levels over the past 3 months. Thus, it is a better indicator of mean blood sugar. Although conflicting results were reported in studies from the same country, this may be due to differences in the sample size as well as the duration of lockdown and social distancing at the time of the survey.

These conflicts can all be related to the differences in the number of participants, times of studies, the lockdown policies, socioeconomic status, and the study locations. Even the results of studies from one continent or country were various. For example, we found that in all Japanese studies, patients gained weight during the lockdown and social distancing (Da Silva et al., 2020; Munekawa et al., 2020; Kishimoto et al., 2021; Tanaka et al., 2021; Aldukhayel, 2022; Maruo et al., 2022; Takahara et al., 2022). Capaldo et al. (2020) represented a decrease in PPBS (1.04%), while Caruso et al., 2021 reported an increase in PPBS (1.45%) during the lockdown and social distancing. These two studies showed different results among participants in Italy (Capaldo et al., 2020; Caruso et al., 2021).

Nutrition guidelines have explained diabetic patients’ eating patterns based on their type, age, and personal characteristic. These guidelines have been flexible, and there have been many similarities between eating patterns for different types of diabetes. In our evaluation, the studies stated that although proper nutritional guidelines were recommended to the patients during the lockdown, they could not follow these guidelines due to lockdown policies and social isolation. We found that most included studies were done on all types of diabetes with no related separate data analysis according to type of diabetes. Also, due to the heterogeneity between studies in evaluated outcomes, the severity and duration of the lockdown policies, and the methodology, we could not pool the results of studies and conduct a meta-analysis.

This systematic review has some strengths and limitations. First, this is the first systematic review to assess the impact of eating habits during the COVID-19 lockdown and social distancing on patients with diabetes. Second, numerous studies were reviewed from all over the world, most of which were rated as satisfactory or good quality studies by the NOS. The limitation of this study is that most studies use self-reported online surveys to assess eating habits. However, the lack of standard and unique practice tools to identify changes in the diet may lead to biased results. We also have the same limitations with measuring glycemic and anthropometric indices in different studies. The included studies have different designs and methods making it difficult to draw firm and pool conclusions about changes in eating habits modification and due to the heterogeneity of designs and methods of studies, we could not conduct a meta-analysis. Besides, internet availability is limited in low-income communities hindering the collection of their health data. There was no statistical comparison of the differences in glycemic indices and dietary patterns between different types of diabetes in the studies. Hence, further studies are recommended to assess the variability of changes in glycemic indices and eating habits between diabetes types.

## Conclusion

The present systematic review revealed that COVID-19 lockdowns and social distancing affect the eating habits of patients with diabetes. In most documents, diabetic patients consumed more fruits, vegetables, and grains and cooked at home while cravings for takeout, fast foods, and alcoholic beverages were reduced. However, the results of the glycemic control were controversial in different studies. Some studies reported the increased BMI and weight possibly owing to consuming more amount and number meals, snacks, and sweets. However, it should be noted that diabetic patients suffer more from the consequences of lockdown and social distancing, such as lack of access to medical supplies, physicians, and healthy food products, as well as psychological stress and less physical activity, hindering the acceptable blood sugar goals. Thus, understanding the changes in eating habits and blood glucose indices in this particular population during the COVID-19 could help the health authorities prepare for future pandemics or other unavoidable global disasters.

## Author contributions

MQ and H-SE came up with the idea of this article and did the final proofreading of the article. FP, ME, NK, and ZG undertook the study search and evaluated the articles. NL and MM wrote the manuscript and the tables. All authors contributed to the article and approved the submitted version.

## Conflict of interest

The authors declare that the research was conducted in the absence of any commercial or financial relationships that could be construed as a potential conflict of interest.

## Publisher’s note

All claims expressed in this article are solely those of the authors and do not necessarily represent those of their affiliated organizations, or those of the publisher, the editors and the reviewers. Any product that may be evaluated in this article, or claim that may be made by its manufacturer, is not guaranteed or endorsed by the publisher.

## Abbreviations

COVID-19: corona virus disease 2019
PRISMA: preferred reporting items for systematic reviews and meta-analyses
FBS: fasting blood sugar
PPBS: postprandial blood sugar
HbA1C: hemoglobin A1C
BMI: body mass index
DM: diabetes mellitus
Type1/2 DM: type 1 or 2 diabetes mellitus
GDM: gestational diabetes mellitus
