# Supplementary material for: How did lockdown and social distancing policies change the eating habits of diabetic patients during the COVID-19 pandemic? A systematic review
Source: Front Psychol. 2022 Sep 23;13:1002665. doi: 10.3389/fpsyg.2022.1002665 (PMC9540372; doi:10.3389/fpsyg.2022.1002665)
Supplement: Supplementary file 1 [file Data_Sheet_1.docx]

**Supplementary Table 1. Newcastle-Ottawa Scale adapted for cross-sectional studies**

| First author, (Year) | Study design | Selection | | | | Comparability | | Outcomes | | Quality score |
| --- | --- | --- | --- | --- | --- | --- | --- | --- | --- | --- |
|  |  | Representativeness of the sample | Sample size | Non-respondents | Ascertainment of the exposure | Data/ results adjusted | Data/results not adjusted | Assessment | Statistical test |  |
| Maruo Y.,et al. (2021) | Cross-sectional  study | 1 | 1 | 1 | 0 | 1 | 0 | 1 | 1 | Satisfactory |
| Sankar P.,et al. (2020) | Cross-sectional  study | 1 | 1 | 1 | 0 | 1 | 0 | 1 | 1 | Satisfactory |
| Olickal J.,et al. (2020) | Cross-sectional study | 1 | 1 | 1 | 0 | 1 | 0 | 1 | 1 | Satisfactory |
| Ghosh A.,et al. (2020) | Cross-sectional study | 0 | 1 | 1 | 0 | 0 | 0 | 0 | 1 | Unsatisfactory |
| Khader M.,et al. (2020) | Cross-sectional  study | 1 | 1 | 1 | 1 | 0 | 0 | 1 | 1 | Satisfactory |
| Tanaka n.,et al. (2021) | Cross-sectional  study | 1 | 1 | 1 | 1 | 0 | 0 | 1 | 1 | Satisfactory |
| Sisman p.,et al. (2021) | Cross- sectional  study | 1 | 1 | 1 | 0 | 1 | 0 | 1 | 1 | Satisfactory |
| Tiwari a.,et al. (2021) | Cross- sectional study | 0 | 1 | 1 | 1 | 1 | 0 | 1 | 1 | Satisfactory |
| Verma m.,et al. (2021) | Cross-sectional  study | 1 | 0 | 1 | 1 | 1 | 0 | 1 | 1 | Satisfactory |
| Carvalhal m.M,et al. (2021) | Cross- sectional  study | 1 | 1 | 1 | 0 | 1 | 0 | 0 | 0 | Unsatisfactory |
| Munekawa c.,et al. (2020) | Cross-sectional study | 1 | 1 | 1 | 0 | 1 | 0 | 1 | 1 | Satisfactory |
| Kishimoto m.,et al. (2021) | Cross-sectional  study | 1 | 1 | 1 | 0 | 1 | 0 | 1 | 1 | Satisfactory |
| Takahara m.,et al. (2021) | Cross-sectional  study | 1 | 1 | 1 | 0 | 1 | 0 | 1 | 1 | Satisfactory |

**Supplementary Table 2. Newcastle-Ottawa Scale adapted for cohort studies**

| First author, (Year) | Study design | Selection | | | | Comparability | | Outcomes | | | Quality score |
| --- | --- | --- | --- | --- | --- | --- | --- | --- | --- | --- | --- |
|  |  | Representativeness of the exposed | Selection of the non-exposed | Ascertainment of exposurents | Demonstration that outcome |  |  | Assessment | Follow-up | Median duration of follow-up |  |
| Khare J.,et al. (2020) | Cohort study | 1 | 0 | 1 | 0 | 2 | 1 | | 1 | 1 | Fair |
| Khare J.,et al. (2020) | Cohort study | 1 | 0 | 1 | 0 | 2 | 1 | | 1 | 1 | Fair |
| Ruiz-Roso M.,et al. (2020) | Cohort study | 1 | 0 | 1 | 0 | 2 | 1 | | 1 | 1 | Fair |
| Caruso I.,et al. (2020) | Cohort study | 0 | 0 | 1 | 0 | 2 | 1 | | 0 | 1 | Fair |
| Capald B.,et al. (2020) | Cohort study | 1 | 0 | 1 | 0 | 2 | 1 | | 1 | 1 | Fair |
| Grabia M.,et al. (2020) | Cohort study | 1 | 0 | 1 | 0 | 2 | 1 | | 1 | 1 | Fair |
| Vetrani C.,et al. (2021) | Cohort study | 1 | 0 | 1 | 0 | 2 | 1 | | 1 | 1 | Fair |
| Amataiti T.,et al. (2021) | Cohort study | 0 | 0 | 1 | 0 | 2 | 1 | | 1 | 1 | Fair |
| Hansel B.,et al. (2021) | Cohort study | 1 | 0 | 1 | 0 | 2 | 1 | | 1 | 1 | Fair |
